# Comparative Proteomics Analysis of Gastric Cancer Stem Cells

**DOI:** 10.1371/journal.pone.0110736

**Published:** 2014-11-07

**Authors:** Tamami Morisaki, Masakazu Yashiro, Anna Kakehashi, Azusa Inagaki, Haruhito Kinoshita, Tatsunari Fukuoka, Hiroaki Kasashima, Go Masuda, Katsunobu Sakurai, Naoshi Kubo, Kazuya Muguruma, Masaichi Ohira, Hideki Wanibuchi, Kosei Hirakawa

**Affiliations:** 1 Department of Surgical Oncology, Osaka City University Graduate School of Medicine, Osaka, Japan; 2 Oncology Institute of Geriatrics and Medical Science, Osaka City University Graduate School of Medicine, Osaka, Japan; 3 Department of Pathology, Osaka City University Graduate School of Medicine, Osaka, Japan; University of Alabama at Birmingham, United States of America

## Abstract

Cancer stem cells (CSCs) are responsible for cancer progression, metastasis, and recurrence. To date, the specific markers of CSCs remain undiscovered. The aim of this study was to identify novel biomarkers of gastric CSCs for clinical diagnosis using proteomics technology. CSC-like SP cells, OCUM-12/SP cells, OCUM-2MD3/SP cells, and their parent OCUM-12 cells and OCUM-2MD3 cells were used in this study. Protein lysates from each cell line were analyzed using QSTAR Elite Liquid Chromatography with Tandem Mass Spectrometry, coupled with isobaric tags for relative and absolute quantitation technology. Candidate proteins detected by proteomics technology were validated by immunohistochemical analysis of 300 gastric cancers. Based on the results of LC-MS/MS, eight proteins, including RBBP6, GLG1, VPS13A, DCTPP1, HSPA9, HSPA4, ALDOA, and KRT18, were up-regulated in both OCUM-12/SP cells and OCUM-2MD3/SP cells when compared to their corresponding parent cells. RT-PCR analysis indicated that the expression level of *RBBP6, HSPA4, DCTPP1, HSPA9, VPS13A, ALDOA, GLG1*, and *CK18* was high in OCUM-12/SP and OCUM-2MD3/SP, in compared with the control of parent OCUM-12 and OCUM-2MD3. These proteins were significantly associated with advanced invasion depth, lymph node metastasis, distant metastasis, or advanced clinical stage. RBBP6, DCTPP1, HSPA4, and ALDOA expression in particular were significantly associated with a poor prognosis in the 300 gastric cancer patients. RBBP6 was determined to be an independent prognostic factor. The motility-stimulating ability of OCUM-12/SP cells and OCUM-2MD3/SP cells was inhibited by *RBBP6* siRNA. These findings might suggest that the eight proteins, RBBP6, GLG1, VPS13A, DCTPP1, HSPA9, HSPA4, ALDOA, and KRT18, utilizing comparative proteomics analysis, were perceived to be potential CSC markers of gastric cancer. Of the eight candidate proteins, RBBP6 was suggested to be a promising prognostic biomarker and a therapeutic target for gastric cancer.

## Introduction

Cancer stem cells (CSCs) are defined as a unique subpopulation in tumors that possess the ability to initiate tumor growth and sustain self-renewal [1]. It has been proposed that they can cause the heterogeneous lineage of cancer cells that constitute the tumor as well as play an important role in the malignant progression of carcinoma, such as distant metastasis, recurrence, and chemoresistance [2]–[4]. CSCs were initially identified in acute myeloid leukemia [5], but have recently been reported to exist in a wide variety of cancers, including gastric cancer [6]. The identification of CSC markers may open a new therapeutic perspective on the basis of selectively targeting this small population of cells [7], [8]. Recently, it has been reported that CSCs possibly do express their own unique markers, such as aldehyde dehydrogenase 1 (ALDH1) [9], CD44 [10], [11], and CD133 [12]. However, many of the published markers are not unique to CSCs. Quantitative protein expression profiling allows efficient identification of accurate and reproducible differential expression values for proteins [13]. Isobaric tags for relative and absolute quantitation (iTRAQ) combined with multidimensional liquid chromatography (LC) and tandem mass spectrometry (LC-MS/MS) analysis is emerging as a powerful methodology in the search for tumor biomarkers [14]. We previously reported that the side population (SP) cells are able to self-renew and produce non-SP cells, and that cancer cells in SP fractions possess high potential for tumorigenicity, distant metastasis[3], and chemoresistance[2]. This suggests that SP cells of gastric cancer possess cancer stem cell-like properties. Therefore, the aim of this study was to detect a novel CSC marker(s) of gastric cancer by comparing the proteomes among parent cells and stem cell-like SP cells that have been known to possess a rich CSC population[15].

## Materials and Methods

### Cell Cultures

Two gastric cancer cell lines, OCUM-2MD3 [16] and OCUM-12 [17], were used in this study. These cell lines were derived from diffuse-type gastric cancer. The culture condition was cultivated in Dulbecco's modified Eagle medium (DMEM; Nikken, Kyoto, Japan) with 10% heat-inactivated fetal calf serum (FCS; Life Technologies, Grand Island, NY), penicillin and streptomycin, and 0.5 mM sodium pyruvate, and incubated at 37°C. OCUM-12/SP and OCUM-2MD3/SP cell lines were SP cells that were evaluated by a flow cytometric analysis using Hoechst 33342 from their parent cell lines, OCUM-2MD3 and OCUM-12, respectively. Sorting was performed three times to establish a stable population of SP-enriched cells. After a three month incubation period post-sorting, OCUM-12/SP cells (6.5%) and OCUM-2MD3/SP cells (12.2%) still represented a high percentage of the SP fraction, compared to parent OCUM-12 (3.2%) and OCUM-2MD3 (6.3%) cells (**Figure S1**). Subsequently, these SP-enriched cells with a stable population were the cell lines used for the analysis, as previously reported [18].

### Human Tissue Specimens and Patient Information

Tissue specimens were obtained from 300 patients diagnosed with gastric cancer permitted operations at Osaka City University. **Table 1** shows the clinicopathologic characteristics of the 300 gastric cancer patients. There were 208 male and 92 female patients, with the median age of 64 years (range, 28–85 years) at the time of operation. The diagnoses were confirmed by at least two people. Staging was determined in accordance with the Japanese classification of gastric carcinoma (14^th^ edition) [19]. This study was approved by the Osaka City University Ethics Committee (Osaka, Japan). Written informed consent from the donor was obtained for use of this sample in research.

**Table 1:** Clinicopathological characteristics of 300 gastric cancer patients.

Clinicopathological features		Number
Sex	Female	92
	male	208
Age	>60	97
	≤60	203
Macroscopic type	Type-4	33
	Other types	267
Tumor differentiation	Intestinal type	154
	Diffuse type	146
Depth of tumor invasion	T1	137
	T2	32
	T3	24
	T4	107
Lymph node metastasis	Negative	165
	Positive	135
Stage	I	146
	II	49
	III	44
	IV	61
Total number of resected lymph node	≤29	155
	≥30	145
Surgery type	D1	141
	D2	159

### Protein Identification and Quantification by QSTAR Elite LC-MS/MS

The cancer cells (60 µg each) were homogenized and then lysed using either 100 µL of T-PER lysis buffer (Thermo Scientific) or 500 µL of 9 M Urea, and 2% CHAPS lysis buffer with a protease inhibitor. Subsequently, the cell lysate was then treated by ultrasonication. After acetone precipitation, protein concentrations were measured by BCA Protein Assay (Pierce, IL, USA). Reduction, alkylation, digestion, and subsequent peptide labeling of 50 µg of protein for each sample were performed using the AB Sciex iTRAQ Reagent Multi-Plex Kit (AB Sciex, Concord, ON, Canada) [20]. The iTRAQ-labeled samples were loaded onto an ICAT cation exchange cartridge (AB Sciex). The peptides were eluted as six fractions (1 mL KCL solution of 10, 50, 70, 100, 200, and 350 mM), and the supernatant of each was evaporated within a vacuum centrifuge. Samples were then desalted and concentrated using Sep-Pak Light C18 cartridges (Waters Corporation, Milford, MA), evaporated within a vacuum centrifuge, resuspended in 20 µL of 0.1% (v/v) formic acid, and subsequently applied onto QSTAR Elite LC-MS/MS. Each sample was run for 150 minutes. MS/MS data was searched against the Swiss Protein database (HUMAN) using ProteinPilot software (version 2.0, AB Sciex) with trypsin set as the digestion enzyme and methyl methanethiosulfinate as the cysteine modification. In order to remove redundant hits and comparative quantitation, the search results obtained were further processed by ProteinPilot software using the Paragon Algorithm. This resulted in the minimal set of justifiable identified proteins. All reported data was used with a 95% confidence cut-off limit. Relative quantitation of peptides was calculated as a ratio by dividing the iTRAQ reporter intensity. The ratios of peptides that support the existence of one protein were averaged for the relative protein quantitation. Thereafter, the ProteinPilot analysis and Ingenuity pathway analysis (IPA) (Ingenuity System, Mountain View, CA) were performed. After performing a Simple t-test on one of the calculated averaged protein ratios against 1 to assess the validity of the protein expression changes, a p-value was reported. Protein ratios with a p-value of less than 0.05 were considered reliable. It should be known that in 90% of the iTRAQ experimental runs done previously, the standard deviations of the protein ratios, which stem from technical variations, were reported to be less than 0.3. Therefore, expression changes greater than 1.2-fold or less than 0.8-fold of normalized expression levels were considered to be outside the range of technical variability. We also performed a non-labeled analysis, and detected the presence of proteins only within OCUM-12/SP cells and OCUM-2MD3/SP cells, but not within parent cells [21]. Each sample was run twice. The applied LC-MS/MS examination coupled with iTRAQ technology have been reported as a reliable quantitative method for protein expression, being even more sensitive than the western blot which depends on the type of applied antibodies [22].

### IPA and Selection of Candidate Proteins

The IPA database is primarily used in the field of proprietary ontology, containing up to 300,000 biological articles including genes, proteins, molecular and cellular processes. Therefore, IPA was employed for the analysis of protein molecular functions, localization. In addition, detailed information regarding the functions and cellular locations of the identified proteins was obtained. Based on the results of LC MS/MS and IPA analyses, proteins that were observed to be over-expressed in SP cell-lines, when compared to their corresponding frequency of expression in parent cell-lines, were selected as candidate biomarkers for SP cells of gastric cancer. The identification of networks of interacting proteins, as well as functional groups and pathways was generated by IPA, and the analysis depends on the previously characterized associations.

### Quantitative Real-time Reverse Transcription-polymerase Chain Reaction (RT-PCR)

Gastric cancer cells were cultured. And the total cellular RNA was extracted using RNeasy Mini Kit (QIAGEN, Carlsbad, CA). cDNA was prepared from 2 µg RNA using random primers (Invitrogen). To determine fold changes in each gene, RT-PCR was performed on the ABI Prism 7000 (Applied Biosystems, Foster City, CA), with commercially available gene expression assays (Applied Biosystems) for *RBBP6* (*retinoblastoma binding protein 6*; Hs00544663), *HSPA4* (*heat shock 70kDa protein 4*; Hs00382884), *HSPA9* (*heat shock 70 kDa protein 9*; Hs00269818), *GLG1* (Golgi glycoprotein 1; Hs00939452), *DCTPP1* (*dCTP pyrophosphatase 1*; Hs00225433), *VPS13A* (*vacuolar protein sorting 13 homolog A*; Hs00362891), *CK18 (keratin 18*; Hs028277483), *ALDOA (aldolase A*; Hs00605108), *CD44* (Hs01075862), *CD133* (Hs01009250) and *NANOG* (Hs02387400). GAPDH (SIGMA) was used as an internal standard to normalize mRNA levels. The threshold cycle (Ct) values were used to calculate the relative expression ratios between control and treated cells.

### Western blot analysis

Expression level of RBBP6 and ALDOA in cancer cells was examined as follows. Cell lysates were collected after different treatments. After the protein concentration of each sample was adjusted, electrophoresis was carried out using 10% Tris/Gly gels (Life Technologies, Carlsbad, CA). The protein bands obtained were transferred to an Immobilon-P Transfer membrane (Amersham, Aylesbury, UK). Then, the membrane was placed in PBS-T solution containing anti-RBBP6 (WH0005930M1, Sigma-aldrich, MO, USA), anti-ALDOA (HPA004177, ATLAS), and anti-β-actin (1∶300 dilution; Sigma-aldrich), and allowed to react at room temperature for 2 hours. The levels of specific proteins in each lysate were detected by enhanced chemiluminescence using ECL plus (Amersham) followed by autoradiography.

### Small Interfering RNA Design

The sequences for *RBBP6* small interfering RNA (siRNA) are designed as follows: si*RBBP6* sense, 5′-GAAAGAAGAAUAUACUGAUtt-3′; antisense, 5′- AUCAGUAUAUUCUUCUUUCgt-3′, and nontargeting siRNA (negative-siRNA) was purchased from Ambion (Life Technologies, Carlsbad, CA).OCUM-12/SP and OCUM-2MD3/SP cells were prepared at 60% confluence in six-well dishes. The transfection mixture was prepared by adding 150 µL of Opti-MEM including 9 µL of Lipofectamine RNA iMAX Regant (Life Technologies) to 150 µL of Opti-MEM including 90 pmol of siRNA and incubating for 5 min at room temperature. Finally, the above transfection mixture was added to prepared six-well dish. Twenty-four hours after transfection, RT-PCR was performed.

### Wound-healing Assay

Cancer cells were cultured in 96-well plates (Essen Instruments, Birmingham, UK). After the cells reached semi-confluence, a wound was created in the cell monolayer with the 96-well by WoundMaker (Essen Bioscience, MI, USA). Scratched fields were taken pictured every 3 hours and was monitored with Incucyte Live-Cell Imaging System and software (Essen Instruments). The degree of cell migrations was analyzed 24 hours after wound treatment as a percentage of wound confluence. The mean of 4 fields was calculated as the sample value.

### Invasion Assay

We used the chemotaxicell chambers (Kubota, Osaka, Japan) with a 12-µm pore membrane filter coated with 50-µg Matrigel (Collaborative Research Co., Bedford, MA). The chamber (upper component) was placed in a 24-well culture plate (lower component). Gastric cancer cells were re-suspended to a final concentration of 5×10^3^ cells/mL. Next, 500-µL lower components. After incubation for 48 h, cancer cells on the upper surface of the membrane were removed by wiping and stained with hematoxylin. Cancer cells that invaded through a filter coated with Matrigel into the lower membrane were manually counted under a microscope at ×200 magnification. Six randomly chosen fields were counted for each assay. The mean of four fields was calculated as the sample value. For each group, the culture was done in triplicate.

### Validation of Protein Expression by Immunohistochemistry

Immunohistochemistry was performed on formalin-fixed, paraffin-embedded tissue samples that were deparaffinized in xylene and dehydrated through graded ethanol. The sections were heated for 10 minutes at 105°C by autoclave in Target Retrieval Solution (DAKO). The samples were subsequently incubated with 3% hydrogen peroxide to block endogenous peroxidase activity. The following antibodies were used in the immunohistochemical process: anti-RBBP6 (retinoblastoma binding protein 6; WH0005930M1, 6∶1000; Sigma-Aldrich), anti-GLG1 (Golgi glycoprotein 1; HPA010815, 1∶80; ATLAS), anti-VPS13A (vacuolar protein sorting 13 homolog A; NBP1-85642, 1∶500; Novus Biologicals), anti-ALDOA (aldolase A, fructose-bisphosphate; HPA004177, 1∶400; ATLAS), anti-DCTPP1 (dCTP pyrophosphatase 1; HPA002832, 1∶200; ATLAS), anti-HSPA9 (heat shock 70 kDa protein 9; HPA000898, 1∶200; ATLAS), anti-HSPA4 (heat shock 70kDa protein 4; HPA010023, 1∶200; ATLAS), and anti-KRT18 (keratin 18; ab668, 1∶500; Abcam). The samples were incubated with each antibody overnight at approximately 4°C. Thereafter, samples were incubated in appropriated immunoglobulin G for 10 minutes, followed by three washes with PBS. All samples were then treated with streptavidin-peroxidase reagent, and incubated in PBS diaminobenzidine and 1% hydrogen peroxide (vol/vol), followed by counterstaining with Mayer's hematoxylin.

### Immunohistochemical Evaluation

RBBP6, GLG1, VPS13A, DCTPP1, HSPA9, HSPA4, ALDOA, and KRT18 expression levels were evaluated by both intensity of staining and proportion of stained tumor cells. The staining intensity was scored on a scale of 0-3 (0 = no, 1 =  mild, 2 =  moderate, 3 =  intense). Staining proportions were scored on a scale of 0–4 (the percentage was different with each antibody) based on the percentage of positively stained cells. Therefore, the final staining score, which was calculated as a multiple of the staining intensity score and the staining proportion score, would be on a scale of 0–12. Expression levels of DCTPP1 were considered positive when it received a score of 3. Expression levels of HSPA4 were considered positive when it received a score of 6. Expression levels of ALDOA, KRT18, VPS13A, and GLG1 were considered positive when each received a score of 8. RBBP6, the evaluation of which only the staining proportion score was used for calculation, was considered positive when it received a score of 3. HSPA9, the evaluation of which only the staining intensity score was used for calculation, was considered positive when it received a score of 3. All evaluations were made by two observers who were unaware of clinical data and outcome. When a discrepant evaluation between the two independent observers was found, the slides were rechecked and reevaluated after discussion.

### Statistical Analysis

The SPSS software program (SPSS Japan, Tokyo, Japan) was used for data analysis. Statistical significance of the associations between the expression of proteins and the various clinicopathological variables, including age, sex, macroscopic type, tumor differentiation, total number of resected lymph node, and type of surgery (D1 or D2 gastrectomy) was evaluated using Fisher's and Chi-squared tests. Survival curves were calculated from the day of surgery to the time of death or to the last follow-up observation using the Kaplan-Meier Method. Additionally, any differences between survival curves were assessed using the Log-rank Test. Multivariate analyses were performed according to the Cox Regression Model to determine the associations between clinicopathological variables and mortality. P-values of <0.05 was considered statistically significant.

## Results

### The stemness of gastric cancer cell lines

The percentages of SP cells were higher in the OCUM-12/SP and OCUM-2MD3/SP cells than in their parent OCUM-12 and OCUM-2MD3 cells (**Figure S1**). Cancer stem cell markers of SP cells, OCUM-12/SP and OCUM-2MD3/SP, such as CD44, CD133, and NANOG, were analyzed by RT-PCR. The expression level of these markers was significantly increased in both SP cell lines, in comparison with their parent cell lines (**Figure S2**). The number of spheroid colony was significantly higher in both OCUM-12/SP and OCUM-2MD3/SP cells than their parent OCUM-12 and OCUM-2MD3 cells (data not shown).

### Detection of Candidate Proteins

**Figure 1:**
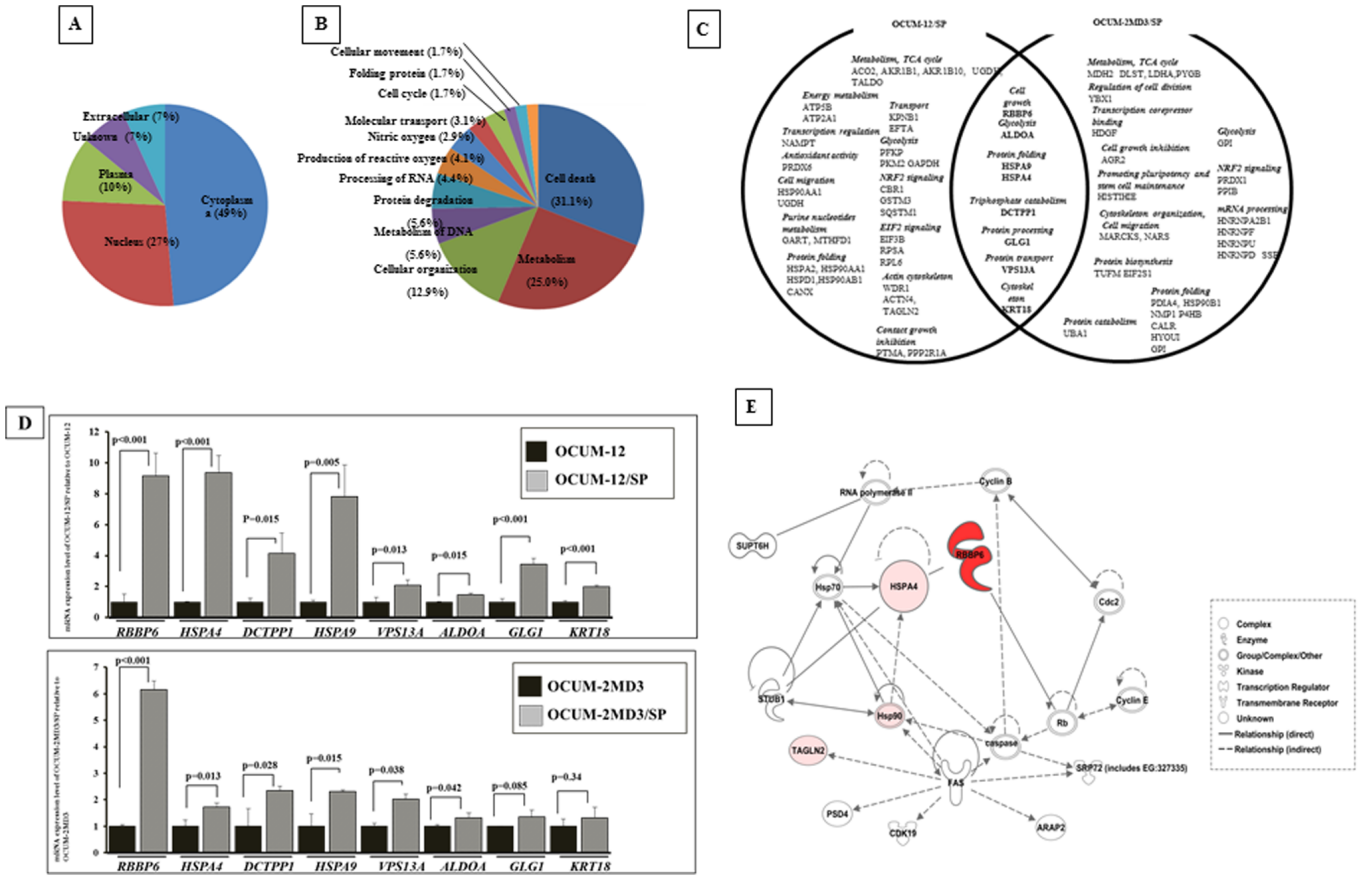
Ingenuity Pathway Analysis (IPA) of Proteins Expressed in SP Cells. (A) Localization; (B) Biological processes of identified proteins. We categorized the proteins based on their functional assignments using Ingenuity Pathway Analysis. The molecular functions reported include cell death, metabolism, cellular organization, metabolism of DNA, protein degradation, processing of RNA, production of reactive oxygen species, nitric oxide, molecular transport, cell cycle, folding protein, and cellular movement. % = 100 X number of identified proteins/all 932 proteins analyzed. (C), A Venn diagram confer to Table 2. Forty proteins were significantly increased in OCUM-12/SP cells compared to their parent OCUM-12 cells. Thirty-five proteins were significantly increased in OCUM-2MD3/SP cells compared to their parent OCUM-2MD3cells. Eight candidate proteins, RBBP6, HSPA4, HSPA9, GLG1, DCTPP1, VPS13A, CK18 and ALDOA, overlap in both OCUM-12/SP and OCUM-2MD3/SP cells. (D), mRNA expression. RT-PCR analysis indicated that the expression level of *RBBP6, HSPA4, DCTPP1, HSPA9, VPS13A, ALDOA, GLG1*, and *CK18* was high in OCUM-12/SP (9.15 fold, 9.36 fold, 4.14 fold, 7.80 fold, 2.08 fold, 1.46 fold, 3.44 fold, and 1.99 fold, respectively) and OCUM-2MD3/SP (6.15 fold, 1.71 fold, 2.33 fold, 2.30 fold, 2.03 fold, 1.32 fold, 1.35 fold, and 1.31 fold, respectively), in compared with the control of parent OCUM-12 and OCUM-2MD3. (E), Correlation of Signaling Pathways between RBBP6 and Differentially-Expressed Proteins in CSC-like SP cells. RBBP6 is over-expressed (red) in CSC-like SP cells (OCUM-12/SP cells and OCUM-2MD3/SP cells). Hsp90, HSPA4, and TAGLN2 (pink) up-regulated in CSC-like SP cells were associated with the RBBP6 signaling pathway.

We investigated whether proteins were differentially or independently expressed in SP cells, and compared our findings to those of their parent cells using QSTAR Elite LC-MS/MS. In analyzing biological processes, and with a 95% confidence cut-off limit and p<0.05, we identified that proteins were indeed differentially expressed. The results of these findings are presented in **Figure 1**. Most of the proteins were over-expressed in the cytoplasm of tumor cells (**Figure 1A**). The P value included in the ingenuity analysis is stated in **Table S1**. These proteins were determined to be related to cellular processes, such as cell death, metabolism, cellular organization, DNA metabolism, protein degradation, and processing of RNA (**Figure 1B**). The top canonical pathways associated with these targets and identified by IPA are shown in **Table S2**.

When compared to their corresponding parent cells, 40 proteins were up-regulated in OCUM-12/SP, and 35 proteins were up-regulated in OCUM-2MD3/SP. Among these proteins, eight were up-regulated in both OCUM-12/SP cells and OCUM-2MD3/SP cells, whereas no such association was observed between their corresponding parent cells (**Table 2** **and** **Figure 1C**). Of these eight proteins, the three proteins, RBBP6, GLG1, and VPS13A, were independently detected in both SP cell lines, but not in their corresponding parent cells. The five proteins, DCTPP1, HSPA9, HSPA4, ALDOA, and KRT18, were significantly over-expressed by at least 1.2-fold in both SP cells when compared to their corresponding parent cells.

**Table 2:** Proteins increased in both OCUM-12/SP and OCUM-2MD3/SP cells compared to their parent cells detected by QSTAR Elite LC/MS/MS.

Symbol	Protein Name	GI Number	UniProt/Swiss-Prot	Ratio^a^	p value	Loca-tion^c^	Type^d^
OCUM-12/SP				OCUM-12/SP			
RBBP6	retinoblastoma binding protein 6	74762440	Q7Z6E9	↑^b^	NA	N	En
GLG1	golgi glycoprotein 1	218512060	Q92896	↑	NA	C	O
VPS13A	vacuolar protein sorting 13 homolog A (S. cerevisiae)	71152975	Q96RL7	↑	NA	C	Tp
DCTPP1	dCTP pyrophosphatase 1	74733624	Q9H773	2.258	0.0206	C	En
HSPA9	heat shock 70 kDa protein 9 (mortalin)	21264428	P38646	1.511	0.0006	C	O
HSPA4	heat shock 70 kDa protein 4	206729934	P34932	1.187	0.009	C	O
ALDOA	aldolase A, fructose-bisphosphate	113606	P04075	1.588	<0.0001	C	En
KRT18	keratin 18	125083	P05783	1.329	0.0003	C	O
NAMPT	nicotinamide phosphoribosyltransferase	1172027	Q4LAY1	1.805	0	ES	cytokine
ACO2 (includes EG:11429)	aconitase 2, mitochondrial	6686275	Q99798	1.549	0.0312	C	En
AKR1B1	aldo-keto reductase family 1, member B1 (aldose reductase)	113596	P15121	1.906	0.0021	C	En
AKR1B10	aldo-keto reductase family 1, member B10 (aldose reductase)	20531983	O83905	1.946	0	C	En
CBR1	carbonyl reductase 1	118519	P16152	1.472	0.0456	C	En
GAPDH	glyceraldehyde-3-phosphate dehydrogenase	120649	P04406	1.095	0	C	En
GART	phosphoribosylglycinamide formyltransferase, phosphoribosylglycinamide synthetase, phosphoribosylaminoimidazole synthetase	131616	P22102	1.254	0.036	C	En
GSTM3	glutathione S-transferase mu 3 (brain)	21264423	P21266	1.304	0.0018	C	En
HSP90AA1	heat shock protein 90 kDa alpha (cytosolic), class A member 1	92090606	P07900	1.25	0.0022	C	En
HSP90AB1	heat shock protein 90 kDa alpha (cytosolic), class B member 1	17865718	P08238	1.487	0.0068	C	En
HSPD1	heat shock 60 kDa protein 1 (chaperonin)	129379	P10809	1.569	0	C	En
MTHFD1	methylenetetrahydrofolate dehydrogenase (NADP+ dependent) 1, methenyltetrahydrofolate cyclohydrolase, formyltetrahydrofolate synthetase	115206	P11586	1.212	0.0196	C	En
PRDX6	peroxiredoxin 6	1718024	P30041	1.345	0.015	C	En
TALDO1	transaldolase 1	6648092	P37837	1.202	0.0044	C	En
UGDH	UDP-glucose 6-dehydrogenase	6175086	B1YLK1	1.198	0.0318	N	En
PFKP	phosphofructokinase, platelet	1346355	Q01813	1.338	0.0019	C	K
PKM2	pyruvate kinase, muscle	20178296	P14618	1.275	0	C	K
ACTN4	actinin, alpha 4	13123943	I4AQQ0	1.208	0.0011	C	O
CANX	calnexin	543920	P27824	1.612	0.0005	C	O
HSPA2	heat shock 70 kDa protein 2	1708307	P54652	1.396	0.0358	C	O
RPL6	ribosomal protein L6	1350762	Q02878	1.368	0.0027	C	O
TAGLN2	transgelin 2	586000	P37802	1.239	0.0134	C	O
WDR1	WD repeat domain 1	12643636	G2TFZ5	1.358	0.0381	ES	O
PTMA	prothymosin, alpha	135834	P06454	1.156	0.0401	N	O
PPP2R1A	protein phosphatase 2, regulatory subunit A, alpha	143811355	P30153	1.439	0.0003	C	phosphatase
SQSTM1	sequestosome 1	74735628	Q13501	1.877	0.0038	C	TR
EIF3B	eukaryotic translation initiation factor 3, subunit B	218512094	P55884	1.234	0.0229	C	TR
RPSA	ribosomal protein SA	125969	P08865	1.31	0.0017	C	TR
ATP2A1	ATPase, Ca2+ transporting, cardiac muscle, fast twitch 1	12643544	G2TF52	1.058	0.0305	C	Tp
ATP5B	ATP synthase, H+ transporting, mitochondrial F1 complex, beta polypeptide	114549	P06576	1.594	0.0111	C	Tp
ETFA	electron-transfer-flavoprotein, alpha polypeptide	119636	P13804	1.473	0.0003	C	Tp
KPNB1	karyopherin (importin) beta 1	20981701	Q14974	1.188	0.0397	N	Tp
OCUM-2MD3/SP				OCUM-2MD3/SP ratio			
RBBP6	retinoblastoma binding protein 6	74762440	Q7Z6E9	↑	NA	N	En
GLG1	golgi glycoprotein 1	218512060	Q92896	↑	NA	C	O
VPS13A	vacuolar protein sorting 13 homolog A (S. cerevisiae)	71152975	Q96RL7	↑	NA	C	Tp
DCTPP1	dCTP pyrophosphatase 1	74733624	Q9H773	1.467	0	C	En
HSPA9	heat shock 70 kDa protein 9 (mortalin)	21264428	P38646	1.778	0.0005	C	O
HSPA4	heat shock 70 kDa protein 4	206729934	P34932	1.27	0.01	C	O
ALDOA	aldolase A, fructose-bisphosphate	113606	P04075	1.533	0.035	C	En
KRT18	keratin 18	125083	P05783	1.618	0.0066	C	O
MDH2 (includes EG:17448)	malate dehydrogenase 2, NAD (mitochondrial)	215274114	P40926	1.604	0.0244	C	En
PRDX1	peroxiredoxin 1	548453	Q06830	1.334	0.0003	C	En
DLST	dihydrolipoamide S-succinyltransferase (E2 component of 2-oxo-glutarate complex)	206729909	P36957	1.277	0.0483	C	En
PDIA4	protein disulfide isomerase family A, member 4	119530	P13667	1.494	0.0353	C	En
PYGB	phosphorylase, glycogen; brain	20178317	P11216	1.87	0.0071	C	En
NARS	asparaginyl-tRNA synthetase	3915059	O53857	2.239	0.0089	C	En
UBA1	ubiquitin-like modifier activating enzyme 1	24418865	P22314	1.353	0.0467	C	En
P4HB	prolyl 4-hydroxylase, beta polypeptide	2507460	P07237	1.852	0.043	C	En
LDHA	lactate dehydrogenase A	126047	P00338	1.373	0.0014	C	En
PPIB	peptidylprolyl isomerase B (cyclophilin B)	215273869	P23284	1.783	0.0112	C	En
GPI	glucose-6-phosphate isomerase	17380385	P06744	2.023	0.0027	ES	En
SSB	Sjogren syndrome antigen B (autoantigen La)	125985	P05455	1.501	0.0222	N	En
HDGF	hepatoma-derived growth factor	1708157	P51858	2.537	0.0468	ES	growth factor
HYOU1	hypoxia up-regulated 1	10720185	Q9Y4L1	1.643	0.0005	C	O
HSP90B1	heat shock protein 90 kDa beta (Grp94), member 1	119360	P14625	3.233	0.0003	C	O
AGR2	anterior gradient 2 homolog (Xenopus laevis)	67462105	O95994	2.172	0.0107	ES	O
HIST1H1E	histone cluster 1, H1e	121919	P10412	1.562	0.0353	N	O
HNRNPA2B1	heterogeneous nuclear ribonucleoprotein A2/B1	133257	P22626	1.634	0.0059	N	O
HNRNPF	heterogeneous nuclear ribonucleoprotein F	1710628	P52597	1.445	0.0393	N	O
MARCKS	myristoylated alanine-rich protein kinase C substrate	76803798	P29966	2.018	0	PM	O
CALR	calreticulin	117501	P27797	4.181	0	C	TR
YBX1	Y box binding protein 1	54040030	P67809	1.401	0.0297	N	TR
NPM1	nucleophosmin (nucleolar phosphoprotein B23, numatrin)	114762	P06748	1.32	0.0201	N	TR
HNRNPD	heterogeneous nuclear ribonucleoprotein D (AU-rich element RNA binding protein 1, 37kDa)	13124489	Q14103	1.483	0.0068	N	TR
TUFM	Tu translation elongation factor, mitochondrial	1706611	P49411	2.205	0.0143	C	TR
EIF2S1	eukaryotic translation initiation factor 2, subunit 1 alpha, 35kDa	124200	P05198	1.474	0	C	TR
HNRNPU	heterogeneous nuclear ribonucleoprotein U (scaffold attachment factor A)	254763463	Q00839	1.637	0.0252	N	Tp

a ratio, OCUM-12/SP ratio: OCUM-12/SP cell compared with parent OCUM-12 cell. OCUM-2MD3/SP ratio: OCUM-2MD3/SP cell compared with parent OCUM-2MD3 cell.

b ↑, proteins expressed only in SP cells but not parent cells.

c Location: C, cytoplasm; PM, plasma membrane; ES, extracellular space; N, nucleus.

d Type: En, enzyme; K, kinase; Tp, transporter; O, other; TR, transcriptional regulator.

NA, not available.

The mRNA expression level of these 8 candidate molecules, *RBBP6, HSPA4, DCTPP1, HSPA9, VPS13A, ALDOA, GLG1*, and *CK18* was increased in OCUM-12/SP (9.15 fold, 9.36 fold, 4.14 fold, 7.80 fold, 2.08 fold, 1.46 fold, 3.44 fold, and 1.99 fold, respectively) and OCUM-2MD3/SP (6.15 fold, 1.71 fold, 2.33 fold, 2.30 fold, 2.03 fold, 1.32 fold, 1.35 fold, and 1.31 fold, respectively) cells, in comparison with those of the control of parent OCUM-12 and OCUM-2MD3 cells (**Figure 1D**). Western blot analysis indicated that the expression level of RBBP6 and ALDOA was high in CUM-12/SP and OCUM-2MD3/SP cells, in comparison with that OCUM-12 and OCUM-2MD3 cells (**Figure S3**).

The network presented in **Figure 1E** was generated by IPA, and the analysis depends on the previously characterized and reported protein interactions. Thus, RBBP6, which was observed to be over-expressed in CSC-like SP cells, OCUM-12/SP cells and OCUM-2MD3/SP cells, was directly related to HSPA4 and Rb, and indirectly associated with Hsp90 and TAGLN2. HSPA4, Hsp90 and TAGLN2 were also found to be up-regulated in CSC-like SP cells.

### Effect of siRBBP6 transfection on the migration and invasive abilities of gastric cancer cells

**Figure 2:**
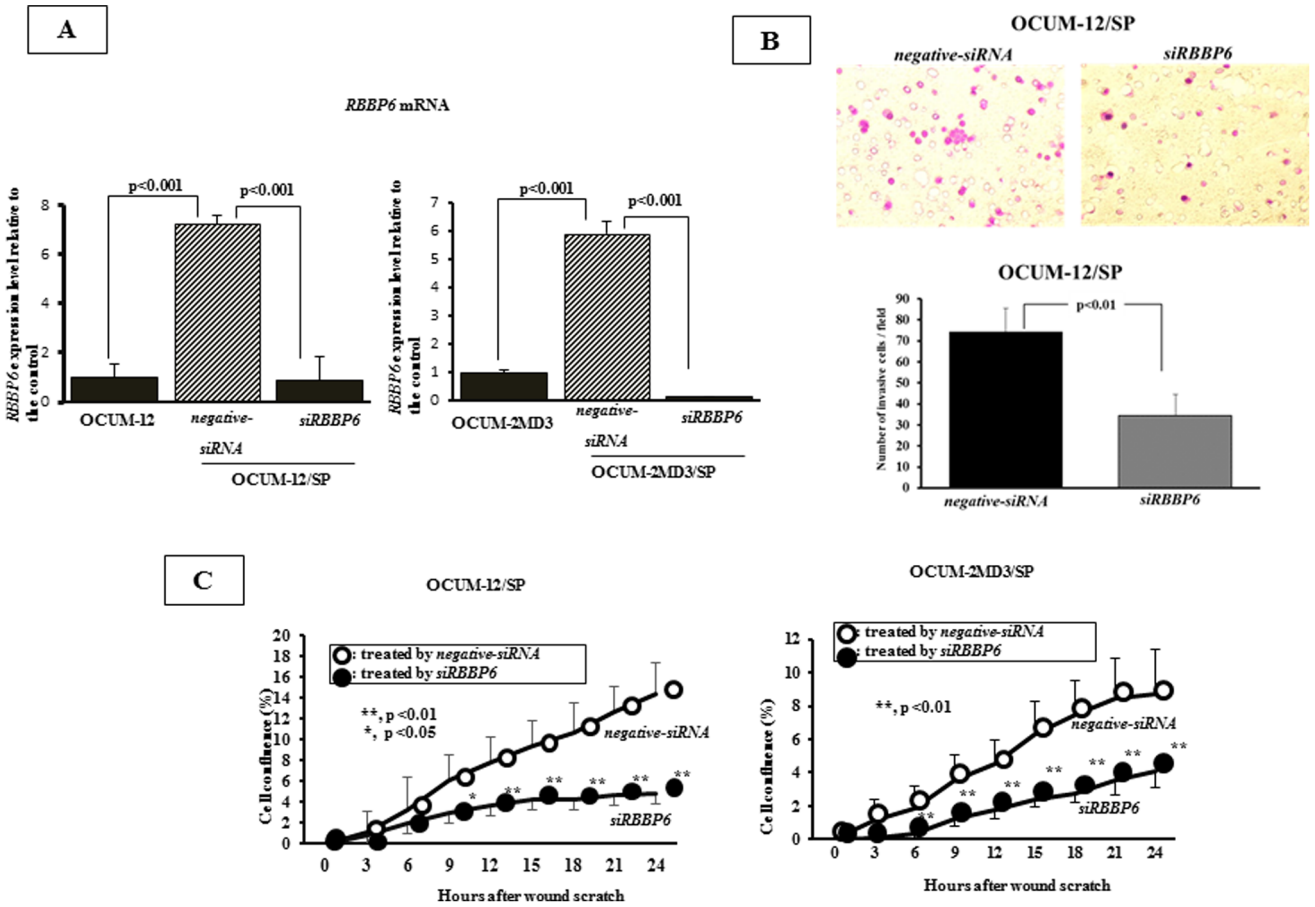
*siRBBP6* transfection into gastric cancer cells. (A), OCUM-12/SP and OCUM-2MD3/SP showed higher level of *RBBP6* mRNA expression than their parent cells, OCUM-12 and OCUM-2MD3. RBBP6 expression in OCUM-12/SP and OCUM-2MD3/SP cells was effectively downregulated by si*RBBP6* transfection. (B), Representative images of invading OCUM-12/SP cells showed the number of cancer cells invading the pore membrane filter was decreased by *RBBP6* siRNA treatment. si*RBBP6* transfection for OCUM12/SP cells significantly inhibited the invasion abilities. Data are presented as the mean and SD (error bars) of four experiments. * *p*<0.05, ** *p*<0.01. (C), si*RBBP6* treatment for OCUM12/SP and OCUM-2MD3/SP cells significantly inhibited the migration abilities, in comparison with that of the control of *negative-siRNA* treatment. Data are presented as the mean and SD (error bars) of four experiments. * *p*<0.05, ** *p*<0.01.

**Figure 2A** shows that si*RBBP6* transfection significantly decreased mRNA expression level of both SP cell lines (OCUM-12/SP was 12%, p<0.01, OCUM-2MD3/SP was 2.5%. p<0.01), in compared with that of negative-siRNA transfection. *RBBP6* siRNA knockdown significantly decreased the invasion (**Figure 2B**) and migration activity (**Figure 2C**) of both SP cells.

### Immunohistochemical Assessment of Candidate Proteins and their Association with Clinicopathological Features

**Figure 3:**
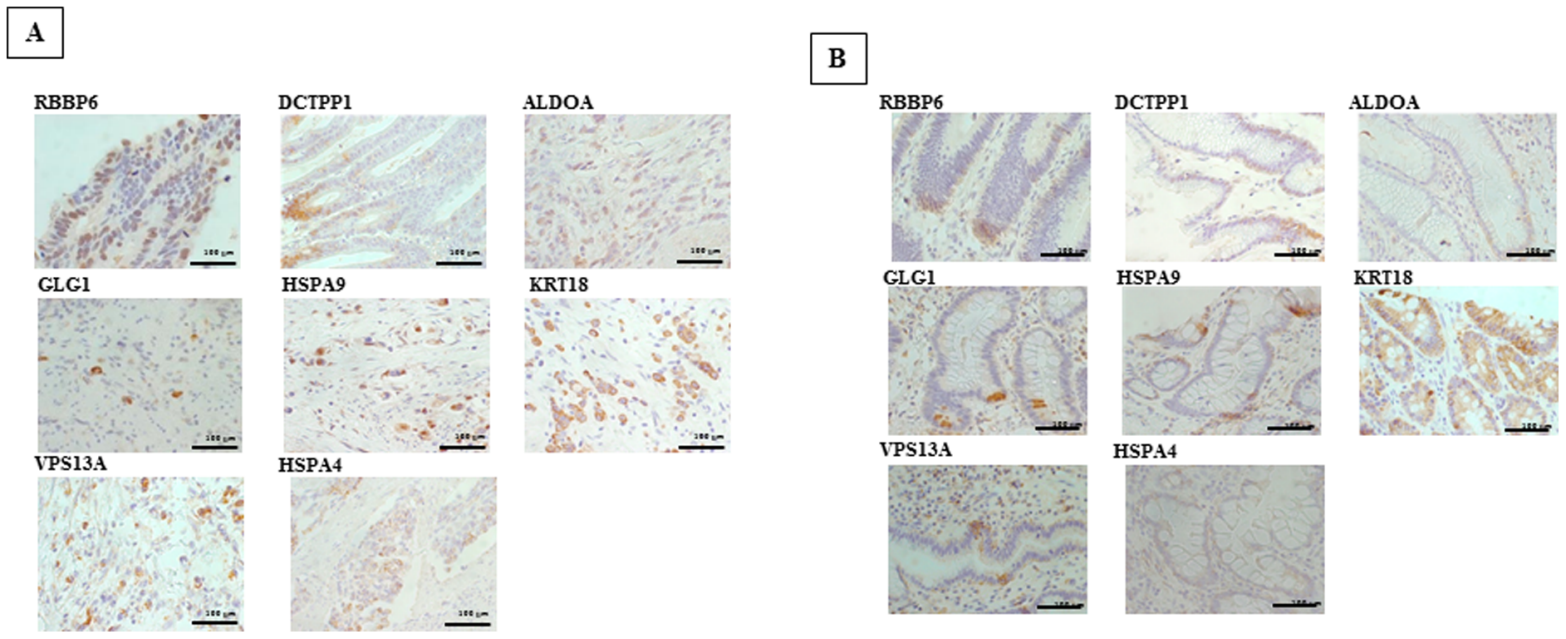
Immunohistochemical Determination. (a) Expression in cancer cells. RBBP6, DCTPP1, and HSPA9 expression were observed primarily in the cytoplasm and nucleus. GLG1, VPS13A, HSPA4, ALDOA, and KRT18 were expressed in the cytoplasm. (b), Expression in epithelial cells. RBBP6, GLG1, VPS13A, DCTPP1, and HSPA9 expression were found some cells in the epithelial gland. KRT18 were expressed in most epithelial cells. HSPA4 and ALDOA expression was not found in normal cells.

RBBP6, DCTPP1, and HSPA9 were observed to be primarily expressed in the cytoplasm and nuclei of gastric cancer cells. GLG1, VPS13A, HSPA9, HSPA4, ALDOA, and KRT18 were observed to be primarily expressed in the cytoplasm (**Figure 3A**). In normal epithelial cells, RBBP6, GLG1, VPS13A, DCTPP1, and HSPA9 expression were found some cells in the epithelial gland. KRT18 were expressed in most epithelial cells. HSPA4 and ALDOA expression was not found in normal cells. BBP6, DCTPP1, and HSPA9 were expressed in the cytoplasm and nuclei of normal epithelial cells. GLG1, VPS13A, HSPA9, and KRT18 were observed in the cytoplasm of epithelial cells (**Figure 3B**).

We explored the association between the expression level of the eight candidate proteins and the clinicopathological features. Number of cases to each score for the eight targets was shown in **Table S3**. These eight proteins were determined to be associated with potentially malignant processes, such as distant metastasis, lymph node (LN) metastasis, invasion depth, or stage advancement (**Table 3**). The calculated p-values were as follows: RBBP6 was significantly associated with invasion depth (p<0.001), LN metastasis (p<0.001), distant metastasis (p = 0.013), and clinical stage (p<0.001); GLG1 was significantly associated with only distant metastasis (p = 0.045). VPS13A was significantly associated with invasion depth (p = 0.005), LN metastasis (p<0.001), and stage advancement (p = 0.005); DCTPP1 was significantly associated with invasion depth (p<0.001), LN metastasis (p<0.001), distant metastasis (p<0.001), and stage advancement (p<0.001); HSPA9 was significantly associated with invasion depth (p<0.001), LN metastasis (p<0.001), and stage advancement (p<0.001); HSPA4 was significantly associated with invasion depth (p<0.001), LN metastasis (p<0.001), distant metastasis (p = 0.007), and stage advancement (p<0.001); ALDOA was significantly associated with invasion depth (p = 0.034), LN metastasis (p = 0.004), and stage advancement (p = 0.029); and KRT18 was significantly associated with invasion depth (p = 0.001), LN metastasis (p = 0.001), distant metastasis (p = 0.034), and stage advancement (p = 0.004).

**Table 3:** Correlation between proteins expression and clinicopathological variables.

Factors	RBBP6			GLG1			VPS13A			DCTPP1		
	positive	negative	p-value	positive	negative	p-value	positive	negative	p-value	positive	negative	p-value
	(n = 151)	(n = 143)		(n = 70)	(n = 221)		(n = 149)	(n = 147)		(n = 140)	(n = 159)	
T category												
T2-T4	102	58	<0.001	46	115	0.045	93	68	0.005	105	57	<0.001
	(64%)	(36%)		(29%)	(71%)		(58%)	(42%)		(65%)	(35%)	
T1	49	85		24	106		56	79		35	102	
	(37%)	(63%)		(19%)	(81%)		(42%)	(58%)		(65%)	(35%)	
Lymph node metastasis												
positive	86	44	<0.001	34	97	0.493	81	50	<0.001	88	44	<0.001
	(66%)	(34%)		(26%)	(74%)		(62%)	(38%)		(67%)	(33%)	
negative	65	99		36	124		68	97		52	115	
	(40%)	(60%)		(23%)	(77%)		(41%)	(59%)		(31%)	(69%)	
Distant metastasis												
positive	40	21	0.013	15	46	0.912	35	26	0.217	41	20	<0.001
	(66%)	(34%)		(25%)	(75%)		(57%)	(43%)		(67%)	(33%)	
negative	111	122		55	175		114	121		99	139	
	(48%)	(52%)		(24%)	(76%)		(48%)	(52%)		(42%)	(58%)	
Clinical Stage												
I	55	89	<0.001	26	114	0.116	58	87	0.005	44	103	<0.001
	(38%)	(62%)		(19%)	(81%)		(40%)	(60%)		(30%)	(70%)	
II	23	24		14	33		31	16		26	22	
	(49%)	(51%)		(30%)	(70%)		(66%)	(34%)		(54%)	(46%)	
III	33	9		15	28		25	18		29	14	
	(79%)	(21%)		(35%)	(65%)		(58%)	(42%)		(67%)	(33%)	
IV	40	21		15	46		35	26		41	20	
	(66%)	(34%)		(25%)	(75%)		(57%)	(43%)		(67%)	(33%)	

Factors	HSPA9			HSPA4			ALDOA			KRT18		
	positive	negative	p-value	positive	negative	p-value	positive	negative	p-value	positive	negative	p-value
	(n = 143)	(n = 151)		(n = 169)	(n = 128)		(n = 77)	(n = 218)		(n = 127)	(n = 163)	
T category												
T2-T4	96	65	<0.001	109	52	<0.001	50	111	0.034	84	76	0.001
	(60%)	(40%)		(68%)	(32%)		(31%)	(69%)		(53%)	(47%)	
T1	47	86		60	76		27	107		43	87	
	(35%)	(65%)		(44%)	(56%)		(20%)	(80%)		(33%)	(67%)	
Lymph node metastasis												
positive	79	52	<0.001	97	34	<0.001	45	86	0.004	72	59	0.001
	(60%)	(40%)		(74%)	(26%)		(34%)	(66%)		(55%)	(45%)	
negative	64	99		72	94		32	132		55	104	
	(39%)	(61%)		(43%)	(57%)		(20%)	(80%)		(35%)	(65%)	
Distant metastasis												
positive	36	25	0.069	44	17	0.007	21	40	0.096	34	27	0.034
	(59%)	(41%)		(72%)	(28%)		(34%)	(66%)		(56%)	(44%)	
negative	107	126		125	111		56	178		93	136	
	(46%)	(54%)		(47%)	(53%)		(24%)	(76%)		(44%)	(56%)	
Clinical Stage												
I	49	94	<0.001	60	86	<0.001	27	117	0.029	48	91	0.004
	(34%)	(66%)		(41%)	(59%)		(19%)	(81%)		(35%)	(65%)	
II	31	16		32	15		13	34		19	28	
	(66%)	(34%)		(68%)	(32%)		(28%)	(72%)		(40%)	(60%)	
III	27	16		33	10		16	27		26	17	
	(63%)	(37%)		(77%)	(23%)		(37%)	(63%)		(60%)	(40%)	
IV	36	25		44	17		21	40		34	27	
	(59%)	(41%)		(72%)	(28%)		(34%)	(66%)		(56%)	(44%)	

### Prognosis

**Figure 4:**
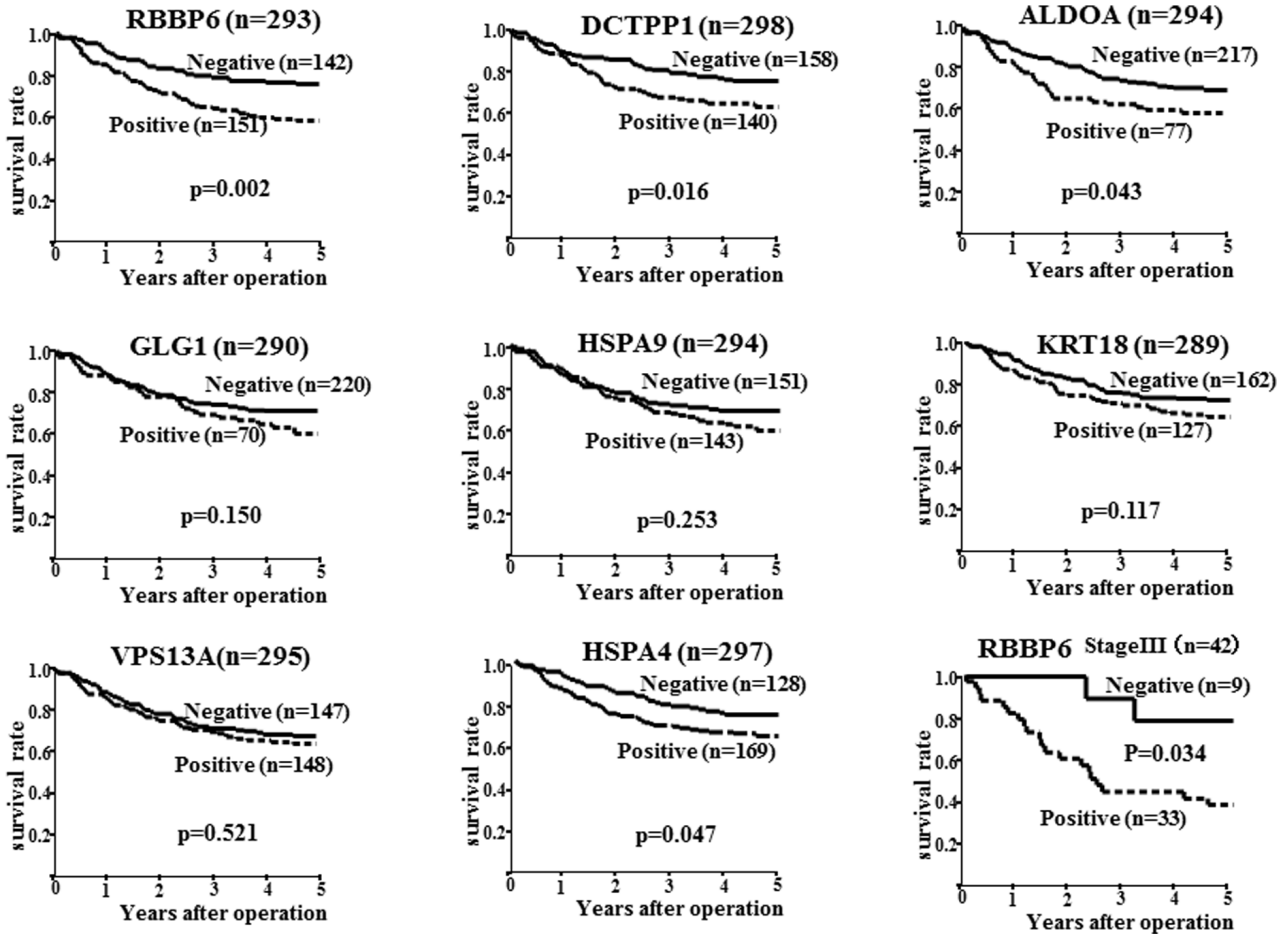
Overall Survival of Patients with Gastric Cancer. The overall survival of patients with gastric cancer in relation to the expression of RBBP6, GLG1, VPS13A, DCTPP1, HSPA9, HSPA4, ALDOA, and KRT18. The prognosis of patients with RBBP6, DCTPP1, HSPA4, and ALDOA expression was significantly poorer than those without expression in gastric cancer cases. The overall survival rate of RBBP6-positive cases was significantly less than that of RBBP6-negative cases in patients at stage III. In contrast, no significant correlations were observed between other proteins and patient survival.

The Kaplan-Meier plots suggested that of the eight over-expressed proteins, RBBP6, DCTPP1, HSPA4, and ALDOA, were significantly associated with poor survival in all patients (**Figure 4**). The cumulative five-year overall survival rate of RBBP6-positive cases (61%) was significantly less (p = 0.002) than that of RBBP6-negative cases (78%). Moreover, in patients at stage III, the overall survival rate of RBBP6-positive cases was significantly less (p = 0.034) than that of RBBP6-negative cases. The prognosis of patients with DCTPP1-positive tumors (63%) was significantly poorer (p = 0.016) than that of DCTPP1-negative tumors (75%). The five-year overall survival rate of HSPA4-positive cases (66%) was significantly less (p = 0.047) than that of HSPA4-negative cases (75%). The five-year overall survival rate of ALDOA-positive tumors exhibiting over-expression (61%) was significantly poorer (p = 0.043) than that of ALDOA-negative tumors (72%). In contrast, no significant correlations were observed between other proteins and patient survival. Following univariate analysis, RBBP6, DCTPP1, HSPA4, and ALDOA expression levels were significantly associated with poor prognosis in 300 gastric cancer patients (**Table 4**). In addition, macroscopic type (type 4), histological type (diffuse), T category (T2-4), vessel invasion, infiltration pattern (INF b, c), peritoneal metastasis, and LN metastasis were determined to be significantly associated with a poor prognosis. Multivariate analysis was performed using the Cox Proportional Hazards Model for all significant variables in the univariate analysis. Upon analysis completion, RBBP6 expression (p = 0.023), Borrmann type 4 (p<0.001), peritoneum positive (p = 0.001), LN metastasis (p = 0.003), and hepatic metastasis (p = 0.001) were confirmed as independent factors correlated with survival (**Table 3**). Of the eight proteins, RBBP6 expression was an independent prognostic factor.

**Table 4:** Univariate and multivariate analyses with respect to survival.

Clinicopathological features	Univariate analysis			Multivariate analysis		
	Risk ratio	95% CI	p-value	Risk ratio	95% CI	p-value
RBBP6						
positive vs negative	1.964	1.275–3.025	0.002	1.770	1.080–2.901	0.023
GLG1						
positive vs negative	1.391	0.885–2.186	0.152			
VPS13A						
positive vs negative	1.145	0.885–2.186	0.521			
DCTPP1						
positive vs negative	1.661	1.096–2.516	0.017	0.902	0.558–1.458	0.674
HSPA9						
positive vs negative	0.983	0.747–1.295	0.905			
HSPA4						
positive vs negative	1.544	1.002–2.378	0.049	0.929	0.549–1.571	0.784
ALDOA						
positive vs negative	1.563	1.010–2.421	0.045	1.454	0.866–2.442	0.156
KRT18						
positive vs negative	1.390	0.919–2.101	0.119			
Age						
>60 vs <60	1.424	0.894–2.268	0.137			
Sex						
Male vs female	1.065	0.680–1.667	0.785			
Macroscopic type						
Type4 vs Other types	9.084	5.701–14.476	<0.001	4.894	2.687–8.914	<0.001
Tumor differentiation						
diffuse vs intestinal	1.647	1.088–2.500	0.018	1.155	0.679–1.965	0.595
T category						
T2-4 vs T1	4.479	2.674–7.504	<0.001	1.187	0.568–2.479	0.648
Vessel invasion						
positive vs negative	3.070	1.967–4.793	<0.001	0.961	0.559–1.653	0.886
INF^a^						
c vs a & b	1.782	1.160–2.737	0.008	0.923	0.512–1.666	0.791
Hepatic metastasis						
positive vs negative	7.776	3.369–17.950	<0.001	4.927	1.953–12.429	0.001
Peritoneal metastasis						
positive vs negative	8.209	4.734–14.236	<0.001	3.043	1.600–5.789	0.001
Lymph node metastasis						
positive vs negative	6.315	3.841–10.384	<0.001	2.848	1.419–5.717	0.003
Total number of resected lymph node						
<29 vs>30	0.903	0.600–1.359	0.626			
Surgery type						
D2 vs D1	1.886	0.589–1.333	0.886			

a NF; Infiltration pattern of tumor. The predominant pattern of infiltrating growth into the surrounding tissue is classified as follows; INF a: The tumor shows expanding growth and a distinct border with the surrounding tissue. INF b: This category is between INF a and INF b. INF c: The tumor shows infiltrating growth and an indistinct border with the surrounding tissue.

## Discussion

Gastric cancer results in a poor prognosis because of frequent metastatic processes, such as LN metastasis and peritoneal metastasis [23]. CSCs have been proposed as having an important role in the malignant potential of cancer cells, including distant metastasis and chemoresistance [4]. We have since discovered that SP cells obtained from gastric cancer subjects possess these CSC properties [3]. We confirmed that SP cells utilized in this study express candidate gastric cancer stem cell markers including CD44[24], CD133 [25], and NANOG [26] (**Figure S2**). The spheroid colony formation activity of these SP cells was higher than that of the parent cells [18]. Also, these CSC-like SP cells display chemoresistance to anticancer drugs [2]. These findings have confirmed that OCUM-12/SP cells and OCUM-2MD3/SP cells may represent cancer stem cell-like properties. Since specific markers for gastric CSCs have not been published as of yet, elucidation of the specific signaling pathways and mechanisms underlying the actions of CSCs might improve the prognosis of gastric cancer. In this study, eight candidate CSCs markers were identified by proteomic techniques using LC-MS/MS coupled with iTRAQ technology. Three proteins, RBBP6, GLG1 and VPS13A, were detected only in SP cell lines but not in their respective parent cell lines. In addition, the five proteins, HSPA9, ALDOA, DCTPP1, HSPA4, and KRT18, were over-expressed in both SP cell lines relative to their respective parent cell lines. RT-PCR analysis also indicated that the expression level of *RBBP6, HSPA4, DCTPP1, HSPA9, VPS13A, ALDOA, GLG1*, and *CK18* was high in OCUM-12/SP and OCUM-2MD3/SP, in compared with the control of parent OCUM-12 and OCUM-2MD3. These proteins were suspected to be novel biomarkers for gastric CSCs. This hypothesis was tested by immunohistochemical analysis of 300 gastric cancer cases.

RBBP6 is a nuclear protein, which is a known to be associated and potentially related to p53, and retinoblastoma binding Q protein 1 (RBQ-1) [27]. RBBP6 interacts with the tumor suppressor proteins p53 and Rb, and plays a role in the induction of apoptosis as well as regulating the cell cycle [28], [29]. RBBP6 binds to wild-type p53 proteins but not to p53 mutants [30]. It has also been shown to promote the binding of MDM2 [31], an E3 ubiquitin ligase that targets the p53[32], and to interfere with its ability to transactivate the target genes [33]. Up-regulation of RBBP6 has been strongly correlated with tumor progression in esophageal cancer and cervical cancer [32]. In this study, RBBP6 expression was associated with ‘T’ category cancer with regards to invasion depth, distant metastasis, lymph node metastasis, and clinical stage. Moreover, RBBP6 expression was significantly associated with poor survival in patients at all stages, particularly at stage III, resulting in the conclusion that RBBP6 was an independent factor for survival. We performed the *RBBP6* siRNA knockdown of *RBBP6* gene using OCUM-12/SP cells and OCUM-2MD3/SP cells in this study. *RBBP6* siRNA knockdown significantly decreased the invasion and migration activity of both SP cells, while the sphere forming activity was not different between the negative-siRNA and *RBBP6* siRNA in SP cells (data not shown). IHC analysis also indicated that RBBP6 expression was associated with invasion depth, LN metastasis, distant metastasis, and a poor prognosis in gastric cancer patients. RBBP6 might be a novel biomarker of gastric CSCs for clinical diagnosis. These findings suggested that RBBP6 might be closely associated with malignant potential of cancer stem cells, rather than stem cell phenotype.

GLG1 is known as a cysteine-rich fibroblast growth factor receptor (FGFR) [34]. GLG1 was concluded to be associated with the tumorigenesis of some carcinomas [35] and malignancy of brain tumors [36]. FGFR signaling possesses broad mitogenic and cell survival mechanisms, and is involved in a variety of biological processes, including embryonic development, cell growth, and tumor invasion [34]. As observed in this study, GLG1 was concluded to be significantly associated with T category cancer with regards to invasion depth. It should be noted that GLG1 may be associated with invasion potential of CSCs via FGFR signaling.

Vacuolar protein sorting (VPS) plays a crucial role in the trafficking of molecules between cellular organelles, such as through the trans-Golgi network. The mutation in this gene causes the autosomal disorder characterized by progressive neurodegeneration. A past study found that frameshift mutations of VPS genes, along with loss of expression of VPS13A proteins, are common in gastric cancers with high microsatellite instability and suggests that these alterations may contribute to the development of cancer [37]. VPS is involved in cancer-related cellular mechanisms, such as proliferation [38], [39]. As observed in this study, VPS13A was expressed in SP cells and was concluded to be associated with a T category cancer and lymph node metastasis of gastric cancer. It should be noted that VPS13A may be associated with the invasion potential of CSCs.

DCTPP1 is expressed in the nucleus of various cancer cells. Zhang et al. suggested that the accumulation of DCTPP1 in the nucleus of tumor cells might be sufficient for maintaining proper DNA replication needed in order to fulfill the requirement for survival and proliferation of the cells [40]. In conclusion, DCTPP1 was determined to be significantly associated with metastatic activity. It should be noted that DCTPP1 may be associated with the DNA replication of CSCs.

HSPA9 and HSPA4 were concluded to be associated with the invasion and metastatic activity of gastric cancer. These two proteins are members of the heat shock protein (HSP)-70 family of chaperones, and HSPA9 is the major protein in the mitochondria. Some studies have suggested that HSPA9 and HSPA4 are relevant to cellular apoptosis and promoting proliferation[41], ; HSPA9 is over-expressed in colon and hepatocellular carcinomas [43], [44], while HSPA4 is over-expressed in breast, colon, ovarian, and pancreatic cancers [45]. The HSPA4/HSPA14 axis induces the migration, invasion, and transformation of cancer cells [46]. HSP27 regulates EMT processes and NF-κB activity to contribute to the maintenance of breast CSCs [47]. Inhibition of the HSP70 protein reduced adhesion and induced apoptosis of both acquired and *de novo* drug resistant cancer cells [48]. The HSPA9 protein is one of the markers of a colon cancer stem cell population [49]. In conclusion, these findings suggest that HSPA9 and HSPA4 are associated with CSCs properties via chaperones for EMT-associated molecules.

ALDOA, aldolase isozymes, is a key glycolytic enzyme that catalyzes the reversible conversion of fructose-1,6-bisphosphate into glyceraldehyde 3-phosphate and dihydroxyacetone phosphate [50]. Glycolysis is one of the key factors for CSC properties. ALDOA has been expressed in a variety of cancers, such as lung cancer, renal cell and hepatocellular carcinoma [51], [52]. As observed in this study, ALDOA was concluded to be significantly associated with the malignant potential of gastric cancer with regards to invasion depth, LN metastasis, and clinical stage. In addition, these findings suggest that ALDOA may be associated with the glycolysis of CSCs.

KRT18 is a type I intermediate filament and its filament partner is keratin 8 (KRT8). KRT18 is involved in intracellular signaling pathways that regulate cell growth [53]. Fortier et al. currently reported that KRT18/KRT8 is associated with the epithelial-mesenchymal transition (EMT) of cancer cells [53]. EMT is ultimately thought to promote tumor progression through the generation of CSC properties [54], [55]. These findings suggested that KRT18 may be associated with the EMT of CSCs.

We previously reported on the proteomic differential display analysis of normal gastric mucosal tissues and human gastric carcinoma cell lines, including OCUM-12 and OCUM-2MD3 [56]. Nineteen protein spots were observed to be up-regulated in SGC cell lines when compared to normal gastric mucosa tissues by using 2-DE and LC-MS/MS. Among the identified increased spots, two proteins, including UDP-glucose 6-dehydrogenase and the electron transfer flavoprotein subunit alpha, were also up-regulated in OCUM-12/SP cells, as opposed to OCUM-12 cells. Three proteins, including nucleophosmin, peroxiredoxin-1, and elongation factor were up-regulated in OCUM-2MD3/SP cells, as opposed to OCUM-2M cells. Conversely, three proteins, 14-3-3 protein sigma, glucosidase 2 subunit beta, and protein DJ-1, were down-regulated in OCUM-12/SP cells as opposed to OCUM-12 cells in this study. Overall, the eight proteins, UDP-glucose 6-dehydrogenase, electron transfer flavoprotein, nucleophosmin, peroxiredoxin-1, elongation factor, 14-3-3 protein sigma, glucosidase 2 subunit beta, and protein DJ-1 may be associated with CSCs as well. To the best of our knowledge, this is the first proteomic analysis that provides evidence that HSPA9, ALDOA, DCTPP1, HSPA4, KRT18, RBBP6, GLG1, and VPS13A may be candidate CSC markers for gastric cancer. In particular, RBBP6 may be a promising predictive marker for the prognosis of patients with gastric cancer.

## Supporting Information

Figure S1 **Representative picture of side population fraction.** Cancer cells, which disappear in the presence of verapamil (lower panel), are outlined and defined as the SP cells. OCUM-12/SP and OCUM-2MD3/SP cells were sorted as SP cells from each of the parent OCUM-12 and OCUM-2MD3 cells, respectively. The percentages of SP cells were higher in the OCUM-12/SP and OCUM-2MD3/SP cells than in their parent OCUM-12 and OCUM-2MD3 cells.
(TIF)

Figure S2 **Stem cell markers expression.** The expression level of CSC markers, ***CD44*** **, CD133 and** ***NANOG*** was significantly higher in OCUM-12/SP (1.6-, 3.6- and 33.2-fold) and OCUM-2MD3/SP cells (2.1-, 5.9- and 3.6-fold) than that in parent OCUM-12 and OCUM-2MD3 cells.
(TIF)

Figure S3 **Expression of RBBP6 and ALDOA.** The expression level of RBBP6 and ALDOA was high in CUM-12/SP and OCUM-2MD3/SP cells, in comparison with that OCUM-12 and OCUM-2MD3 cells.
(TIF)

Table S1 (DOCX)

Table S2 **Proteins increased in both OCUM-12/SP and OCUM-2MD3/SP cells.**
(DOCX)

Table S3 (DOCX)
