# Supplementary figures and images for: Comparative Proteomics Analysis of Gastric Cancer Stem Cells
Source: PLoS One. 2014 Nov 7;9(11):e110736. doi: 10.1371/journal.pone.0110736 (PMC4224387; doi:10.1371/journal.pone.0110736)

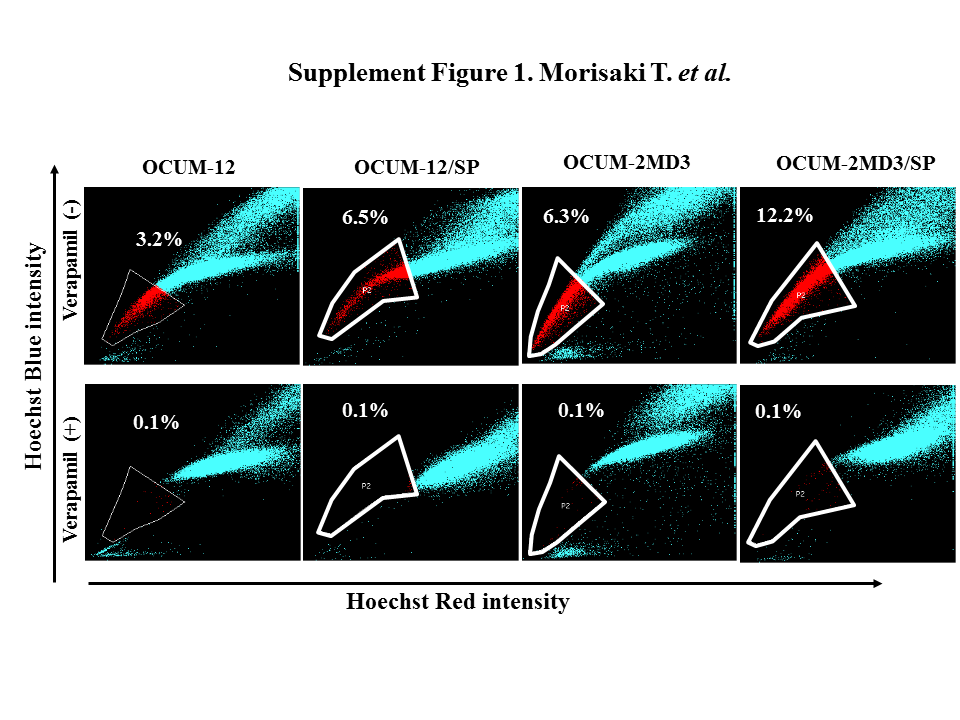

Supplement: Figure S1 — Representative picture of side population fraction. Cancer cells, which disappear in the presence of verapamil (lower panel), are outlined and defined as the SP cells. OCUM-12/SP and OCUM-2MD3/SP cells were sorted as SP cells from each of the parent OCUM-12 and OCUM-2MD3 cells, respectively. The percentages of SP cells were higher in the OCUM-12/SP and OCUM-2MD3/SP cells than in their parent OCUM-12 and OCUM-2MD3 cells. (TIF) [file pone.0110736.s001.tif]
