# Supplementary material for: Comparative Proteomics Analysis of Gastric Cancer Stem Cells
Source: PLoS One. 2014 Nov 7;9(11):e110736. doi: 10.1371/journal.pone.0110736 (PMC4224387; doi:10.1371/journal.pone.0110736)
Supplement: Table S1 — (DOCX) [file pone.0110736.s004.docx]

**Supplement Table 1. Ingenuity pathway analysis (IPA) for each diseases and function annotation**

| Category | Functions Annotation | p-Value |
| --- | --- | --- |
| Cell Death | cell death | <0.001 |
| Metabolism (protein, lipid, carbohydrate, nucleic acid) |  | 0.02 |
| Cellular Assembly and Organization | organization of cytoskeleton | <0.001 |
| DNA Replication, Recombination, and Repair | metabolism of DNA | <0.001 |
| Protein Degradation | degradation of protein | <0.001 |
| RNA Post-Transcriptional Modification | processing of RNA | <0.001 |
| Production of reactive oxygen species |  | 0.012 |
| Production of nitric oxide |  | 0.015 |
| Molecular Transport | transport of protein | 0.01 |
| Cell Cycle | cytokinesis | 0.009 |
| Protein Post-Translational Modification | folding of protein | <0.001 |
| Cellular Movement | cytokinesis | 0.009 |
