# Supplementary material for: Comparative Proteomics Analysis of Gastric Cancer Stem Cells
Source: PLoS One. 2014 Nov 7;9(11):e110736. doi: 10.1371/journal.pone.0110736 (PMC4224387; doi:10.1371/journal.pone.0110736)
Supplement: Table S2 — Proteins increased in both OCUM-12/SP and OCUM-2MD3/SP cells. (DOCX) [file pone.0110736.s005.docx]

**Supplement Table 2. Canonical pathways associated with these targets and identified by ingenuity pathway analysis.**

| Category | p-value | Molecules |
| --- | --- | --- |
| Inflammatory Response | 0.0435 | ENO1,OTUB1,TKT,ANXA1,ALDOA* |
| Carbohydrate Metabolism | 0.0359 | ENO1,FASN,TKT,PGAM1,PHGDH,ALDOA* |
| Cellular Assembly and Organization | 0.0464 | HSPA4*,ANXA1,KRT18*,ALDOA* |
| Cell Death | 0.0359 | HSPA4*,S100A6,ENO1,ANXA1,KRT18*,FASN,HSPA9*,ALDOA*,AARS,S100P |
| Cellular Movement | 0.0435 | S100A6,OTUB1,ANXA1,S100P |
| Lipid Metabolism | 0.0483 | FASN,ANXA1,PHGDH |
| Nervous System Development and Function | 0.0445 | ANXA1,TKT,PHGDH |
| Neurological Disease | 0.0378 | FASN,PHGDH,S100P |
| Small Molecule Biochemistry | 0.0483 | HSPA4*,DCTPP1*,FASN,TKT,ANXA1,PHGDH,ALDOA* |
| Cell Cycle | 0.0359 | FASN,OTUB1 |
| Cell-To-Cell Signaling and Interaction | 0.0397 | KRT18*,OTUB1,FASN,ANXA1 |
| DNA Replication, Recombination, and Repair | 0.0186 | FASN,OTUB1 |
| Energy Production | 0.0359 | FASN,ALDOA |
| Nucleic Acid Metabolism | 0.0118 | DCTPP1*,FASN,TKT,ALDOA* |
| Post-Translational Modification | 0.0445 | HSPA4*,DCTPP1*,OTUB1 |
| Molecular Transport | 0.0225 | ANXA1,HSPA9*,PHGDH,ALDOA* |
| Cell Signaling | 0.0291 | HSPA4*,FASN |
| Protein Synthesis | 0.0445 | DCTPP1* |
| Free Radical Scavenging | 0.0411 | GSR,PARK7,TRAP1,CTTN |

*, Proteins increased in both OCUM-12/SP and OCUM-2MD3/SP cells.
