# Supplementary material for: Comparative Proteomics Analysis of Gastric Cancer Stem Cells
Source: PLoS One. 2014 Nov 7;9(11):e110736. doi: 10.1371/journal.pone.0110736 (PMC4224387; doi:10.1371/journal.pone.0110736)
Supplement: Table S3 — (DOCX) [file pone.0110736.s006.docx]

**Supplement Table 3. Number of cases to each score of gastric tumors.**

|  | Number of cases to IHC score of gastric tumors in each target | | | | | | | |
| --- | --- | --- | --- | --- | --- | --- | --- | --- |
| Score | RBBP6  (n=294) | GLG1  (n=291) | VPS13A  (n=296) | DCTPP1  (n=299) | HSPA9  (n=294) | HSPA4  (n=297) | ALDOA  (n=296) | KRT18  (n=290) |
| 0 | 7 | 16 | 10 | 81 | 1 | 0 | 6 | 8 |
| 1 | 66 | 15 | 9 | 44 | 59 | 21 | 32 | 2 |
| 2 | 70 | 35 | 33 | 34 | 92 | 42 | 37 | 13 |
| 3 | 50 | 13 | 17 | 59 | 142 | 16 | 31 | 0 |
| 4 | 101 | 40 | 34 | 9 | 0 | 50 | 46 | 49 |
| 5 | 0 | 0 | 0 | 0 | 0 | 0 | 0 | 0 |
| 6 | 0 | 102 | 44 | 58 | 0 | 62 | 67 | 91 |
| 7 | 0 | 0 | 0 | 0 | 0 | 0 | 0 | 0 |
| 8 | 0 | 30 | 52 | 0 | 0 | 16 | 32 | 43 |
| 9 | 0 | 24 | 24 | 14 | 0 | 47 | 16 | 39 |
| 10 | 0 | 0 | 0 | 0 | 0 | 0 | 0 | 0 |
| 11 | 0 | 0 | 0 | 0 | 0 | 0 | 0 | 0 |
| 12 | 0 | 16 | 73 | 0 | 0 | 43 | 29 | 45 |
